# Neuromusic research: some benefits of incorporating basic research on the neurobiology of auditory learning and memory

**DOI:** 10.3389/fnsys.2013.00128

**Published:** 2014-02-10

**Authors:** Norman M. Weinberger

**Affiliations:** Department of Neurobiology and Behavior, Center for the Neurobiology of Learning and Memory, Center for Hearing Research, University of CaliforniaIrvine, CA, USA

**Keywords:** auditory cortex, representational plasticity, acetylcholine, gamma oscillations, memory strength

Research on the brain mechanisms of music is currently booming, as it ought. This happy state of affairs is a far cry from early days, when our findings of cortical neurons specifically sensitive to musical contour in the cat had to be sub-divided into two reports of differential emphasis; “contour” was “too musical” for neurophysiologists while cellular discharges were “too neurophysiological” for music researchers (Weinberger and McKenna, 1988; McKenna et al., 1989).

While research on music and the brain continues to burgeon, it seems to make little contact with the larger domain of which it is a part; I refer to the learning and remembering of sounds in general. A vast literature on the basic brain mechanisms of auditory learning and memory (BALM) has developed over the past 25 years (reviewed in Scheich et al., 2011; Weinberger, 2011, 2014). While conducted mainly in animals, the salient findings have been confirmed in humans (e.g., Morris et al., 1998; see also DaCosta et al., 2013). Yet a recent special issue of the *Annals of the New York Academy* (“*The Neurosciences and Music*, *IV*: *Learning and Memory*,” 2012), while authoritative and exciting, includes virtually no reference to the broader context of auditory learning or its neural substrates. In the brief space available here, I hope to attract your interest to BALM by noting potential benefits of incorporating it into studies of neuromusic.

We will consider four central findings, all of which are likely to be critical to understand how the brain learns and remembers sounds, keeping in mind that research to date has focused on relatively simple sounds. Nonetheless, these findings are illustrative of principles of primary auditory cortical processing that overturn the standard concept of A1 as a static feature detector. (1) Receptive field tuning (to frequency and other acoustic parameters) is plastic throughout line span, and can shift toward sounds that become behaviorally relevant. This specific bias for important sounds often increases the area of their representation in the cortex. (2) Such representational plasticity (RP) does not merely serve perception but is a foundation of specific auditory memory. For example, RP has the major attributes of associative memory. It is associative, highly specific, develops rapidly, consolidates (becomes stronger over days) and can be retained indefinitely; the greater the plasticity, the stronger the memory. (3) The direct engagement of muscarinic cholinergic receptors in A1 is *sufficient* to increase the area of representation and to *implant* specific behavioral auditory memory by increasing representational area (e.g., Bieszczad et al., 2013). (4) Both A1 plasticity and the learning of the *behavioral meaning* of sound can be predicted by (and may require) *increased synchronization* of auditory cortical cellular activity (Headley and Weinberger, 2011, 2013); this has been detected in gamma waves (30–120 Hz), which also increase in human cortex in learning.

First, what does it mean to assert that neuromusic research lies within the general domain of BALM? Simply, that remembering heard music, whether “passively” or during the practice of an instrument or voice, is fundamentally the same task as remembering non-musical sounds. This does not imply the same neural substrates, because different auditory cortical fields and networks are likely to be specialized for various parameters of sound. Thus, neurons in primary auditory cortex appear to “prefer” onset transients, but not offset transients. Other regions of human auditory cortex appear to be more greatly involved in processing certain musical and language sounds. Yet others may operate on longer time scales of even tens of seconds. For example, area A2 in the cat auditory cortex is more sensitive than A1 to the omission of a tone within a melody that had been previously presented repeatedly (Weinberger and McKenna, 1988).

But unless completely independent auditory learning systems evolved for music vs. other sounds, the principles underlying acquiring and storing sounds are likely to have a great deal in common. Therefore, findings about the remodeling of auditory cortex in auditory learning are potentially applicable to the learning and remembering of musical sounds. Indeed, the findings of enhanced cortical responses to and enlarged representations of musical sounds (e.g., Pantev et al., 2003; Lappe et al., 2011) were predictable from the BALM literature showing that behaviorally relevant sounds can develop increased responses, gain processing capacity and ultimately increased cortical area (reviewed in Weinberger, 2011).

Research on the *mnemonic functions* of RP in auditory learning has shown that the greater the gain in cortical area, the stronger the memory [(2) above]. Researchers interested in how to facilitate learning and strengthen musical memory could benefit by considering techniques from BALM, even if different than standard music education approaches. For example, stronger memory for melodies might be achieved by using learning strategies that promote their increased representation, such as rewarding their detection or discrimination (perhaps in masked conditions) (Bieszczad and Weinberger, 2010).

Even neural mechanisms of music, such as neurotransmitters and cellular interactions [(3) and (4)], can be studied in humans. For example, cholinergic agents are routinely used in research on memory, so they can easily be exploited in research on the neural bases of music learning and memory. Similarly, psychopharmacological approaches are routinely used to study the involvement of other neuromodulatory systems (adrenergic, dopaminergic and serotonergic) involved in BALM, as well as important excitatory and inhibitory transmitters such as glutamine and GABA, respectively. With respect to neuronal interactions, although invasive recording of cellular discharges is not feasible in humans, the synchronized activity of cortical cells can be detected as gamma band oscillations (~30–120 Hz). Such gamma activity during in humans can accurately predict the strength of later recall (Sederberg et al., 2007; Lenz et al., 2008). The learning of musical sounds, phrases, etc. might be facilitated by beginning with stimuli that evoke strong gamma activity at the outset of training, so that strong musical representations at the start of training could facilitate learning of other musical material.

Of course, caveats apply. BALM studies have concentrated on simple sounds, especially pure tones. This has been done to take advantage of the tonotopic map within A1, thus permitting detection of neural signatures of auditory learning. BALM studies will need to meet neuromusic studies by using more complex acoustic stimuli. But regardless of the extent to which the details of neural mechanisms in learning to hear and remember music transcend those for BALM stimuli used to date, the principle features of specific associative plasticity, neuromodulatory involvement and the coordination of neuronal activity are likely to remain. An incorporation of lessons from animal studies of auditory learning and memory into neuromusic research would recognize that nature apparently does not honor distinctions that neuroscientist often make.
